# Vocalisations as a potential indicator of parturition in C57BL/6J mice

**DOI:** 10.1017/awf.2025.10022

**Published:** 2025-07-07

**Authors:** Sara Capas-Peneda, Ana Ferreira, Colin Gilbert, Jan-Bas Prins, Ashley Vanderplank, Giorgio Rosati, Marco Garzola, Ingrid Anna Sofia Olsson, Gabriela Munhoz Morello

**Affiliations:** 1 i3S – Instituto de Investigação e Inovação em Saúde da Universidade do Porto, Porto, Portugal; 2 ICBAS – School of Medicine and Biomedical Sciences, Universidade do Porto, Porto, Portugal; 3 The Babraham Institute, Cambridge, UK; 4 Leiden University Medical Center, Leiden, The Netherlands; 5 The Francis Crick Institute, London, UK; 6 Tecniplast S. p. A., Buguggiate, Italy

**Keywords:** Animal welfare, breeding, C57BL/6J, home cage monitoring system, laboratory mouse, parturition, vocal communication

## Abstract

Breeding management in laboratory rodents is challenging, particularly around parturition and the neonatal period, where cage disturbance is often avoided in an attempt to limit neonatal mortality. Nevertheless, cage-side observations and single daily checks frequently underestimate pup numbers born and miss parturition complications. Home Cage Monitoring (HCM) systems are gaining popularity in animal facilities, detecting critical events such as food availability and activity levels. Parturition is a complex event involving specific patterns of behaviour, activity and vocalisations. In this study, audio and video data were collected from parturition events of single-housed C57BL/6J females and breeding pairs housed in a prototype rack with integrated microphones. Vocalisations were detected during parturition in both housing conditions, with minimal vocalisations observed prior to parturition, except for ultrasonic sounds in pair-housed mice (*Mus musculus*). After parturition, all vocalisations gradually decreased. Despite limitations such as the need for post-event analysis and the focus on a single mouse strain, this study suggests that detecting vocalisations can be a promising basis for developing automated parturition detection. This highlights the potential of HCM systems for improving breeding management and welfare in laboratory rodent colonies.

## Introduction

Recent advances in technology provide an opportunity to use automated monitoring approaches for fast and accurate detection of critical events in laboratory animal husbandry and welfare, without direct human interference. One such event is parturition in mouse (*Mus musculus*) breeding cages. It is common practice to avoid disturbing cages with females nearing parturition or with newborn litters in an effort to prevent neonatal mortality (The Jackson Laboratory 2009). However, since most pup mortality occurs in the first 48 h and most cadavers are cannibalised, irregular or infrequent cage-side observations are insufficient to accurately detect the onset of parturition, number of pups born and their viability, which leads to poor information on the progress of birth, including an underestimation of true neonatal mortality rates (Brajon *et al.* 2021). Annex III of the EU Directive 2010/63/EU stipulates that laboratory animals must be checked at least daily by a competent person (European Commission 2010). In practice, however, in busy breeding units this often translates as each animals’ cage being checked only once every 24 h. Consequently, birth records can be unreliable. Timely and accurate detection of parturition in animal facilities can be important for research questions related to the study of development or parturition physiology (Robertson *et al.* 2010) and for providing prompt veterinary support for dystocia (Hankenson 2014), improving survival for fragile strains (Strege *et al.* 2024), and reducing pain and suffering.

Home Cage Monitoring (HCM) systems for laboratory rodents are based on technology that allows for the continuous collection of data with minimal human interference on behavioural (activity, social behaviour, learning and memory, feeding) and/or physiological parameters (heart rate or body temperature) (Kahnau *et al.* 2023). There are several commercially available HCM systems such as the Digital Ventilated Cage (Tecniplast, Italy) (Iannello 2019), and Mouse Matrix (Animalab, USA) (Animalab 2024). In addition to providing research data, these systems can also be used for the detection of husbandry-related states, such as food and water availability, changes in animal activity level or the occurrence of unexpected water floods (Iannello 2019; Kahnau *et al.* 2023).

Systems for automated analysis of behavioural parameters rely predominantly on different kinds of tracking technologies (Kahnau *et al.* 2023). Until very recently, these systems were only able to provide information on animals’ movement and position. Although machine learning and artificial intelligence are now enabling the development of automated strategies to assess behaviourally complex events, commercially available technology relies mostly on the collection of video data and retrospective analysis. Nevertheless, some systems allow for real time analysis and identification of behaviours, such as grooming, drinking, feeding, rearing or certain types of stereotypical behaviours (Salem *et al.* 2015; Grieco *et al.* 2021). However, detecting complex behaviours such as play or parturition remains a significant challenge. The introduction of software like DeepLabCut has allowed researchers to customise systems for the real-time detection of complex behaviours (Zahran *et al.* 2024). Yet, to the best of our knowledge, no commercially available systems currently allow for the real-time analysis of complex behaviours, such as parturition, ‘out of the box’ at a scale large enough to monitor an entire animal facility.

Automated parturition detection has been achieved in farm species, such as cattle and small ruminants, using detection of movement with wearable devices such as pedometers or accelerometers, computer vision to detect postures related to parturition (Santos *et al.* 2022), intravaginal implants to detect changes in temperature and the expulsion of the foetus or vulvar magnetic sensors to detect vulvar lip separation during labour (Crociati *et al.* 2022). While some of these approaches are impracticable in small rodents, such as the intravaginal implants or vulvar magnetic sensors, due to their small size and invasiveness, others such as levels of activity could potentially be used in automated HCM systems. Their main limitation is still the individual identification of animals in social contexts (Kahnau *et al.* 2023). Addressing this issue would require either social isolation — which should be avoided as mice are a social species — or the implantation of RFID microchips, a procedure that is both invasive and time-consuming.

So far, little attention has been given to the role of vocalisations in HCM monitoring. Considering the important role that vocal communication plays in mouse reproductive behaviour (Capas-Peneda *et al.* 2022), it may be a candidate for automated detection of key reproductive events. When searching for candidate indicators of parturition, vocalisations associated with the birthing process and with the presence of pups seem particularly relevant. Adult mice are known to vocalise in negative affective states, such as pain and emotional distress (Williams *et al.* 2008; Ruat *et al.* 2022). Pain around parturition is poorly studied in mice, but labour is generally perceived to be painful in mammalian females, primarily as a result of uterine contractions (Martinez-Burnes *et al.* 2021). In parent-offspring interactions post-partum, pup vocalisations play an important role in eliciting maternal responses (Capas-Peneda *et al.* 2022). These include ultrasonic vocalisations (USVs: 30–90 kHz), usually associated with isolation, broadband spectrum signals (4–40 kHz) (Haack *et al.* 1983) which are known to inhibit biting and injury from adults and low-frequency calls (10–20 kHz), often referred to as wriggling calls, which instigate maternal behaviour, such as licking of the pups (Ehret & Bernecker 1986). An additional potentially relevant context is mating behaviour, as laboratory mice are often bred in continuous pair co-housing where post-partum courtship and mating can occur (Berry & Linder 2007). In addition to USVs associated with male courtship behaviour, pup USVs may also indirectly play a role, in that neonatal USVs reduce female aggression and most matings occur during post-partum oestrus, when the female is especially receptive to neonatal USVs (Whitney *et al.* 1973). During courtship, females interact with males through vocalisations in the ultrasonic range while they also produce audible vocalisations (Lupanova & Egorova 2015; Neunuebel *et al.* 2015; Ronald *et al.* 2020).

Analysis of vocalisations is a laborious task that requires specialist knowledge of bioacoustics, a factor that has hampered its widespread adoption in research laboratories. Recent innovations within the domain of machine learning and deep neural networks has rendered vocalisation analysis more accessible, and several tools that facilitate sound analysis have been developed which automate USV detection and analysis, such as VocalMat (Fonseca *et al.* 2021), MUPET (Van Segbroeck *et al.* 2017), Ultravox XT (Noldus 2024), USVSEG (Tachibana *et al.* 2020), A-MUD (Zala *et al.* 2017), HybridMouse (Goussha *et al.* 2021) and, DeepSqueak (Coffey *et al.* 2019). Deepsqueak is an open-source software suite developed to automatically identify and classify vocalisations using deep learning and neural network architecture (Faster-RCNN). It also provides a user-friendly interface for manual reviewing, editing, and labelling of vocalisations. Initially aimed at automating the identification of mouse and rat USVs (Coffey *et al.* 2019), Deepsqueak, with its graphical user interface, allows users to train tailored neural networks for the analysis of vocalisations of different species, such as primates and marine mammals (Romero-Mujalli *et al.* 2021; Ferguson *et al.* 2022), without requiring complex programming skills. It has been described as outperforming previously developed software packages (Binder *et al.* 2021, 2022); however, there is no reference to its use for the analysis of murine audible vocalisations.

In this study, we applied a semi-automated approach using Deepsqueak to identify sound patterns associated with parturition in breeding laboratory mice in their home cage, including both USV and audible vocalisation.

## Materials and methods

### Study animals and housing

Data collection was performed at the Biological Research facility of The Francis Crick Institute (London, UK) between June and December 2022. Three to four months old C57BL/6J male (n = 11) and female (n = 15) mice bred in-house were housed in individually ventilated cages (EM500: 384 × 207 × 145 mm; width × depth × height; Tecniplast, Italy) on an adapted rack. Each cage was provided with aspen chips (Aspen Chips 4, Aspen 4HK, Datesand, UK) bedding material, 10 g of white paper rolls (Enrich-n’Nest©, Datesand, United Kingdom) as nesting material, renewed at each cage change every two weeks. Water and standard food pellets (Teklad global diet [Envigo, UK] autoclaved before use) were provided *ad libitum.* Water bottles were filled with water from pouches made in-house using a Hydropac AWS-2500 pouch machine (PLEXX, The Netherlands). Room temperature was kept at 20–24°C and relative humidity at 45–65%. The animals were maintained on a 12:12 h light regime with lights gradually switched on from 0700h.

**Figure 1.**
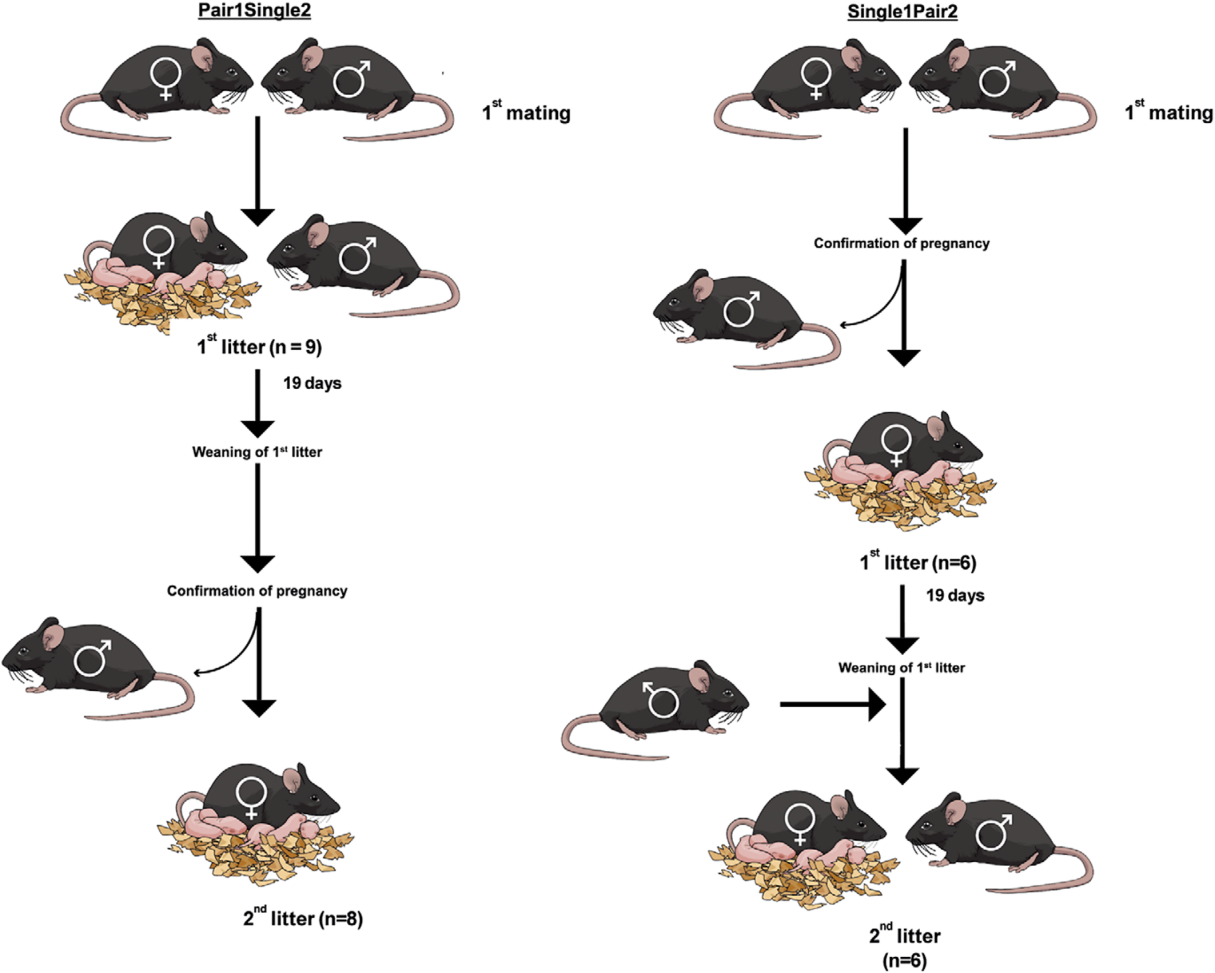
Experimental treatments with indication of numbers of litters of C57BL/6J mice (*Mus musculus*) born in each treatment. In addition, two third litters from single-housed females were also included.

Matings were allowed to occur overnight by co-housing male-female pairs (see Figure 1). The presence of a vaginal plug was verified in the early morning of the day after mating. In order to collect data under single- and pair-housing conditions, the females were either co-housed with the male or the male was removed after plug verification post-mating.

Breeding mice from both groups were identified by shaving the fur on a 3-cm^2^ area on the right thigh of females and the back of males. In addition, the females’ tails were coloured using a red permanent marker and the males’ tails were coloured using a black permanent marker. Pregnant females and their male cage-mate were fur- and tail-marked on day 18 of gestation and re-marked on the tail every two days, avoiding the day of parturition. The marking procedure lasted, on average, 20 s, did not require the use of anaesthetics, and allowed quick recognition of mice by human observers on video recordings during both light and dark phases.

Cages were inspected once a day at 1000 (± 1) h to manually identify the occurrence of new births and count live and dead pups, from birth until day 4 after parturition. Cage inspection was performed by removing the cage from the rack, transferring it to a laminar flow chamber, opening the cage lid and, if necessary, removing the food hopper. Pups were gently touched, if needed.

To avoid litter overlap (which negatively affects newborn survival; Brajon *et al.* 2019), pups were weaned at 19 days after birth. Cages with pups were supplemented with wet mash obtained by mixing feed powder (Teklad global diet) with water in a petri dish placed on the cage floor from day 16 onwards, to ensure that they were nutritionally independent and presented as adequate size at the time of weaning. At weaning, cages were supplemented with wet mash (as described previously) at the bottom of the cage. All animals remained in good body condition after weaning.

### Experimental treatments

Experimental treatments were chosen in order to be able to separate male-female vocal communication from other types of vocalisations. A total of sixteen females were used and randomly allocated to one of two housing groups.

‘Pair1Single2’ females were pair housed for the first litter and single housed for the second. ‘Single1Pair2’ females were single housed for the first litter and pair housed for the second. For the first mating, pairings were carried out by co-housing the male and the female in a new cage. In the Pair1Single2 group, the male and the female remained together until the second pregnancy had been confirmed. In the Single1Pair2 group, the male and female remained together until confirmation of the first pregnancy, and for the second mating, the female was re-introduced into the male cage after weaning of the first litter (Figure 1).

The data were obtained from thirty-one litters from 1st (n = 15) and 2nd (n = 14) litters originating from 15 pair-housed and 16 single-housed breeding females (see Figure 1). Since we had recordings for a third litter for two cages with single-housed females, these were included to help obtain a greater pool of data. At day 18 of pregnancy, cages were moved to the adapted cage positions (see *Equipment*). Recordings were performed from 24 h before birth until 72 h after birth.

### Video and audio recordings

#### Equipment

Three cage positions were adapted for continuous simultaneous visual and sound data recording starting at least 24 h before the expected parturition date until 72 h after the first observation of a litter in the cage. Video recordings were obtained at 30 frames per second through the use of one CCTV bullet camera (DS-2CE17H0T-IT3E, HikVision, China) per cage position, placed in front of the cage connected to a digital video recorder (DS-7204HUHI-K1/P, Hikvision, China). The positioning of the camera, combined with the use of standard nesting material that prevented the formation of dome-shaped nests, enabled the visualisation of all parturitions. Sound recordings were obtained with a sample rate of 250 kHz by a MEMS microphone (VM1000, Vesper, USA) located inside the cage on the back wall.

#### Establishing the exact time of parturition from video recordings

**Figure 2.**
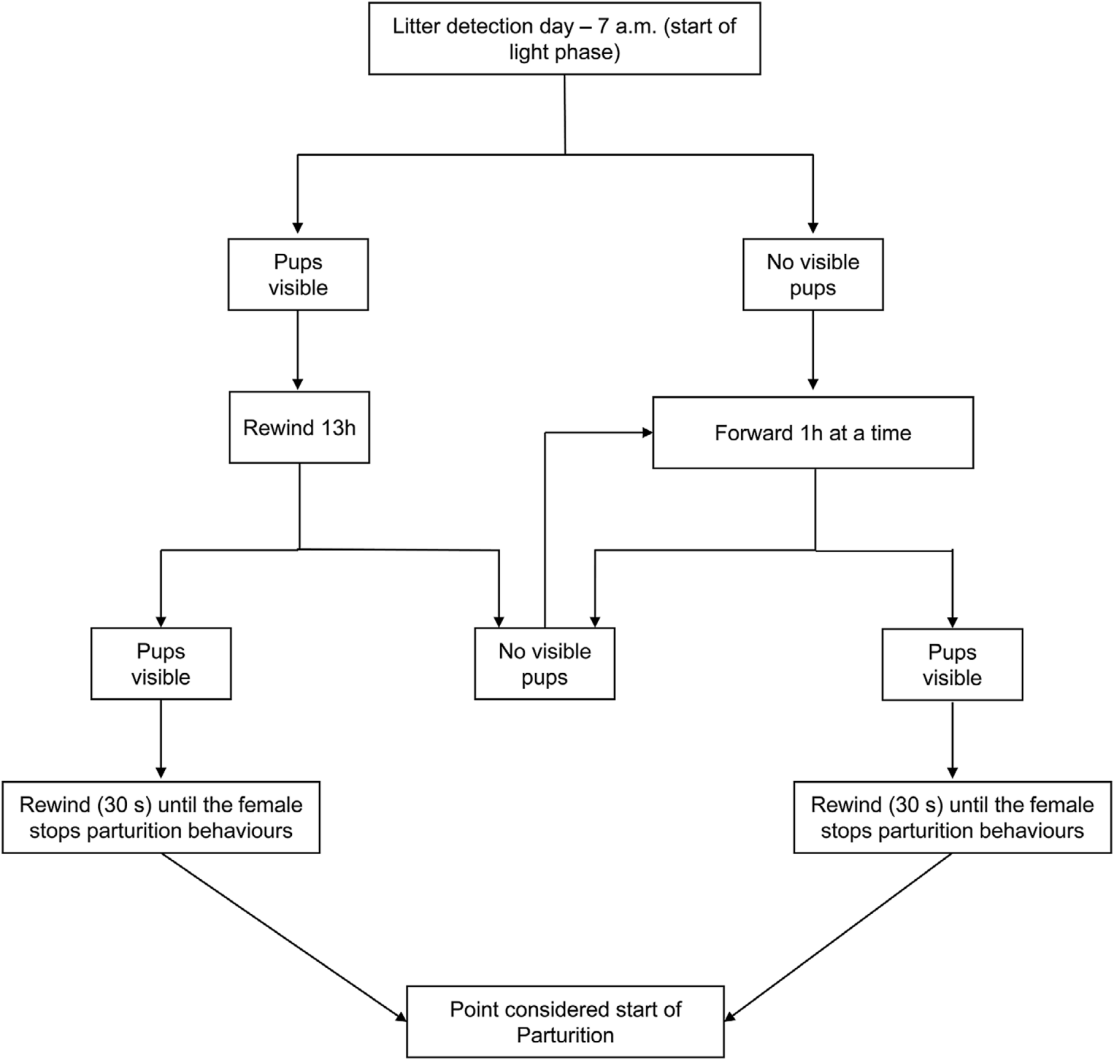
Flowchart describing the different steps to detect parturition in study C57BL/6J mice (*Mus musculus*) from video recordings.

To determine the onset of parturition, video recordings were analysed with BORIS software (version 8.13), starting with identifying the appearance of the first pup. The video was subsequently reviewed backwards (30 s at a time; see Figure 2) until the female was not seen performing parturition-related behaviours (e.g. stretching, circling, arching, and squashing; Ferreira *et al.* 2023). This moment was specified as the commencement of pre-parturient behaviour, as a means of accurately and independently establishing the times of parturition events as a baseline against which to analyse vocalisations.

#### Sound analysis

The audio-recording system was programmed to continuously record sound to cover the entire 24-h periods before and after parturition. At the time, the prototype system used live upload to the cloud storage with only a few hours of internal storage in case of internet outages. During the experiment, there were frequent internet outages which disrupted data storage in the cloud, resulting in random loss of data. Approximately 60% of the stored audio files contained less than 15 min of recordings. To avoid data being highly unbalanced across hours relative to parturition, a scan-sampling approach was applied to the recorded data. One complete 15-min audio file was used per cage per hour relative to parturition for data analysis. An algorithm was devised to find which 15-min period within each of the recorded hours had the greatest number of cages with at least one complete 15-min audio file. Pre-partum hour 18 and post-partum hours 2, 8, and 13 had at least one complete audio file in all 24 cages. Except for hour 24 post-partum, which only had seven cages with at least one complete audio file saved, all studied hours had complete files from at least 20 cages. Cages with more than one complete sound file had one single file selected to represent that specific hour, based on its starting minute; the file closest to the middle of an hour (minute 30 within the hour) was selected. This selection protocol minimised the difference between the selected file starting minute and those of the remaining cages. The resulting sampling approach is compatible with a 15-min scan-sampling method that was applied to all available cages per hour. Therefore, all the audio-data processing and analysis were carried out considering each of the studied hours as represented by one complete 15-min long audio file per cage.

The raw audio files (.raw) were imported into the Audacity software (version 3.4.1) and converted to .wav files using the following settings: Encoding: Signed 16-bit PCM; Byte order: Little-endian; Channels: 1 Channel (Mono); Sampling rate: 250,000 Hz. Converted wav files were manually screened using Raven Lite 2 (Cornell Lab of Ornithology, USA) for the occurrence of vocalisations.

These vocalisations were categorised based on their frequency; above 20 kHz and below 20 kHz; corresponding to the conventional categories USV and audible to humans (henceforth referred to simply as ‘audible’).

The latter were further categorised according to their duration (s) into three groups: short (< 0.03 s), medium (0.03–0.1 s), and long (≥ 0.1 s); these categories were based on visual inspection of the data.

To enhance the efficiency of vocalisation detection, we employed DeepSqueak with a semi-automatic system. DeepSqueak already provides neural networks for the identification of ultrasonic vocalisations but not for audible vocalisations. Hence, a neural network was constructed using files containing 132 calls with background noise and calls present during parturition, the period for which the highest number of audible vocalisations is expected. These calls were manually identified from eleven parturition events randomly selected (comprising both single- and pair-housed conditions).

The network was trained eight times with 24 files (which were not included in the analysis). Subsequently, the trained network was used for semi-automatic detection of vocalisations. Each file was scanned for the identification and classification of the vocalisations present, and thereafter the files were exported in CSV format for further analysis and interpretation.

### Statistical analysis

Number of calls per hour was initially modelled as a function of the fixed effects of type of call (four-level categorical variable: Long, Medium, and Short audible calls, and USVs), period relative to parturition (two-level categorical variable: pre-parturient and parturient/post-parturient, including the 24 h prior to and post the commencement of parturition behaviours, respectively), housing configuration (two-level categorical variable: single- or pair-housed adult breeding mice), and all possible interactions between these variables. However, model residuals failed to be normally distributed and variance homoscedasticity was not achieved. Thus, a preliminary non-parametric Mann-Whitney *U* test was performed by using the NPAR1WAY function on SAS (SAS Institute Inc, USA) considering Wilcoxon scores to compare total number of calls during the pre-parturient and parturient/post-parturient periods. For a more detailed analysis, the response variable ‘number of calls per hour’ was classified into being zero or above zero. Procedure Logistic was used on SAS to evaluate the probability of calls occurring (response variable) as a function of the aforementioned independent variables. Least square means were compared considering a 95% confidence interval.

Number of calls after commencement of parturition behaviours were regressed as a function of number of hours since commencement of parturition behaviour, housing configuration, and type of call. Fundamental frequency (referring to the base frequency of a periodic sound wave at which the vocal folds and respiratory structures vibrate ([Hirst & Looze 2021]) of audible calls was modelled as a function of number of hours post-partum, housing configuration, and type of audible call. A similar approach was taken to analyse the fundamental frequency of the USVs. As USVs were also frequent before parturition, USV fundamental frequency was also modelled as a function of housing configuration and period relative to parturition (two-level categorical variable: before and after the commencement of parturition behaviour), and the interaction between these two variables. All analyses of number of calls after parturition and fundamental frequency were performed by using Procedure GENMOD on SAS with a logarithmic link function. For number of calls during and after parturition, a Poisson data distribution was considered.

## Results

**Figure 3.**
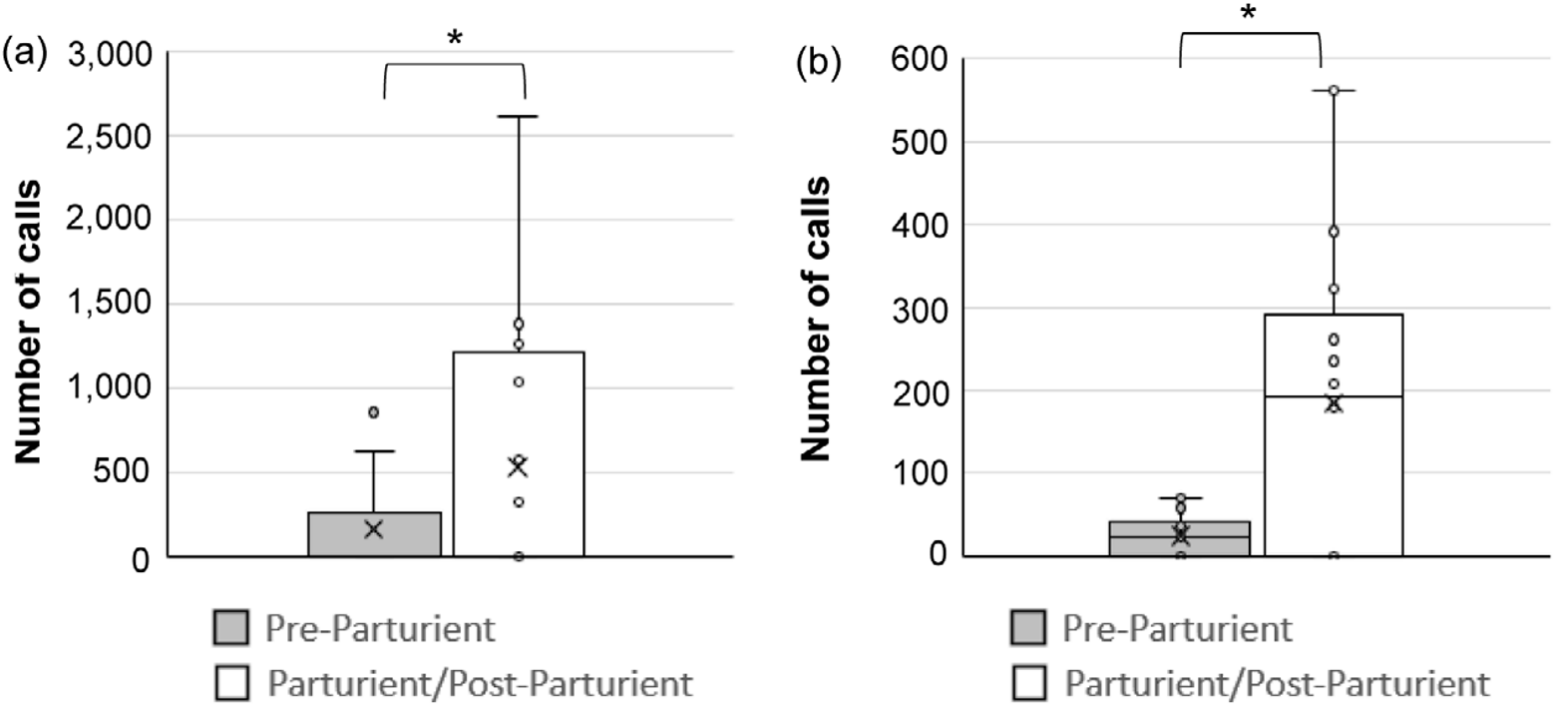
Total number of calls per cage by study C57BL/6J mice (*Mus musculus*) across all pre-parturient (Pre; 24 h prior to commencement of parturition behaviours), and parturient and post-parturient (Part/Post; 24 h post commencement of parturition behaviours) periods in (a) pair- and (b) single-housed cages. Fifteen minutes were analysed per hour. Number of observations: SH Pre: 1,056; SH Part/Post: 1,128; PH Pre: 860; PH Part/Post: 904. * *P* < 0.05.

After commencement of parturition, a large number of calls of all types were detected in both pair- and single-housed adult mice. In contrast, before parturition calls were negligible except for USV in pair-housed mice. Table 1 depicts the total and mean number of calls obtained within each category for pair- and single-housed adult mice, while Figure 3 illustrates box-and-whisker diagrams for total number of calls during the pre-parturient (24 h prior to commencement of parturition behaviours), parturient and post-parturient periods (24 h post commencement of parturition behaviours), with the result of the preliminary non-parametric comparison between these periods.

**Table 1.** Total and mean (± SD) number of audible calls (of long, medium and short durations) and ultrasound vocalisations (USVs) relative to commencement of parturition behaviours (Pre: pre-parturient and Part/Post: parturient and post-parturient, including all 24 h prior to and post commencement of parturition behaviours, respectively), per housing category

		Total number of calls | Mean (± SD) number of calls per hourly 15-min period				Total number of observations
	Call Length Category:	Long		Medium		
PH	Pre	13 |	0.06 (± 0.50)	262 |	1.22 (± 16.03)	215
	Part/Post	406 |	1.80 (± 7.79)	2,416 |	10.69 (± 23.86)	226
SH	Pre	0 |	0.00 (± 0.00)	13 |	0.05 (± 0.38)	264
	Part/Post	259 |	0.92 (± 1.85)	1,870|	6.63 (± 9.02)	282
		Short		USVs		
PH	Pre	25 |	0.12 (± 0.77)	2,981 |	13.87 (± 54.33)	215
	Part/Post	605 |	2.68 (± 8.27)	9,937 |	43.97 (± 140.40)	226
SH	Pre	5 |	0.02 (± 0.31)	161 |	0.61 (± 1.92)	264
	Part/Post	366 |	1.30 (± 1.78)	688 |	2.44 (± 6.01)	282

SH: Single Housed; PH: Pair-housed mice. 15 minutes were analysed per hour. No statistical analysis results are presented here.

**Figure 4.**
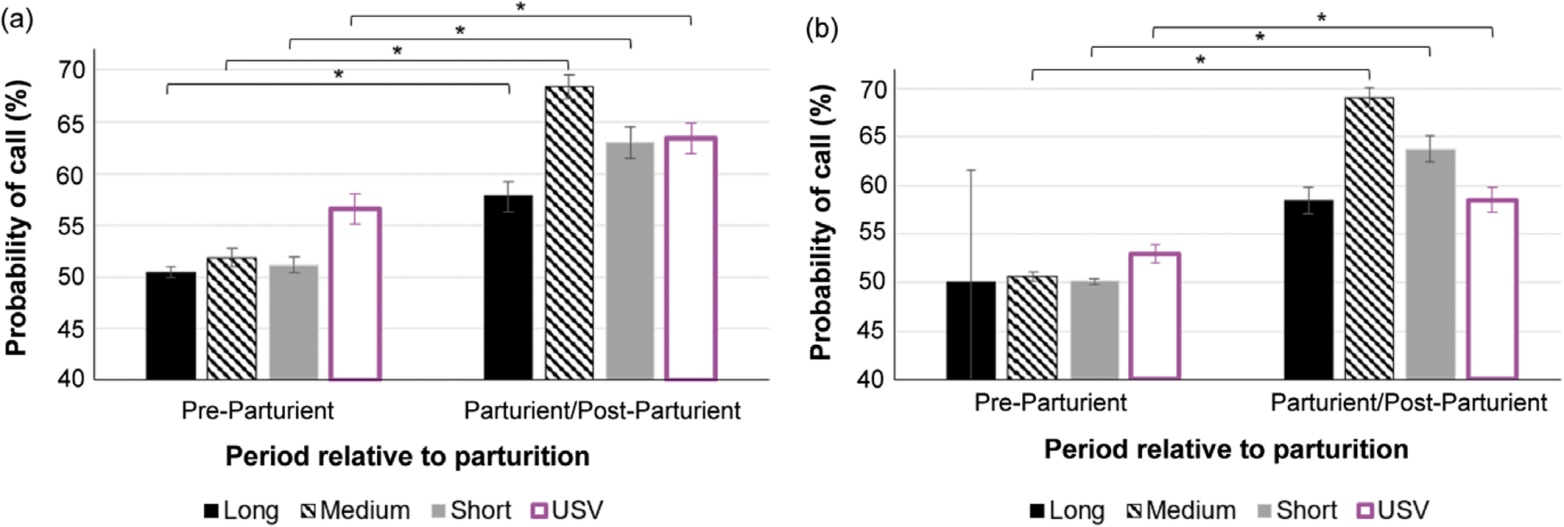
Probability of occurrence of calls (model adjusted means) with standard errors in (a) pair- and (b) single-housed cages of study C57BL/6J mice (*Mus musculus*), per type of call (audible Long, Medium, Short calls, and USVs) and period relative to parturition (Pre-parturient and Parturient/Post-Parturient, including all 24 h prior to and post commencement of parturition behaviours, respectively). Y-axis starts at 50% probability, i.e. calls are equally likely to happen or not. Values above 50% mean calls are more likely to happen. For number of observations, please see Table 1. * *P* < 0.05.

In cages with pair-housed adult mice, the probability of calls occurring was higher after the commencement of parturition behaviour compared to before for Long (Z = 6.32; *P* < 0.001), Medium (Z = 12.32; *P* < 0.001), Short (Z = 8.98; *P* < 0.001) audible calls, and USVs (Z = 5.95; *P* < 0.001). In cages with a single-housed female mouse, the probability of calls occurring was higher after the commencement of parturition behaviour than before for Medium (Z = 11.74; *P* < 0.001) and Short (Z = 5.78; *P* < 0.001) audible calls, and USVs (Z = 6.0; *P* < 0.001). There were no occurrences of Long audible calls before parturition in cages with single-housed mice for comparison with post-partum levels. Model adjusted means are depicted in Figure 4.

The probability of audible (Long, Medium, and Short) calls happening did not differ (*P* > 0.05) between cages with pair- and single-housed adult mice, while USVs were more likely to occur in cages with pair-housed mice both before (Z = 4.06; *P* < 0.001) and after (Z = 4.59; *P* < 0.001) parturition.

**Figure 5.**
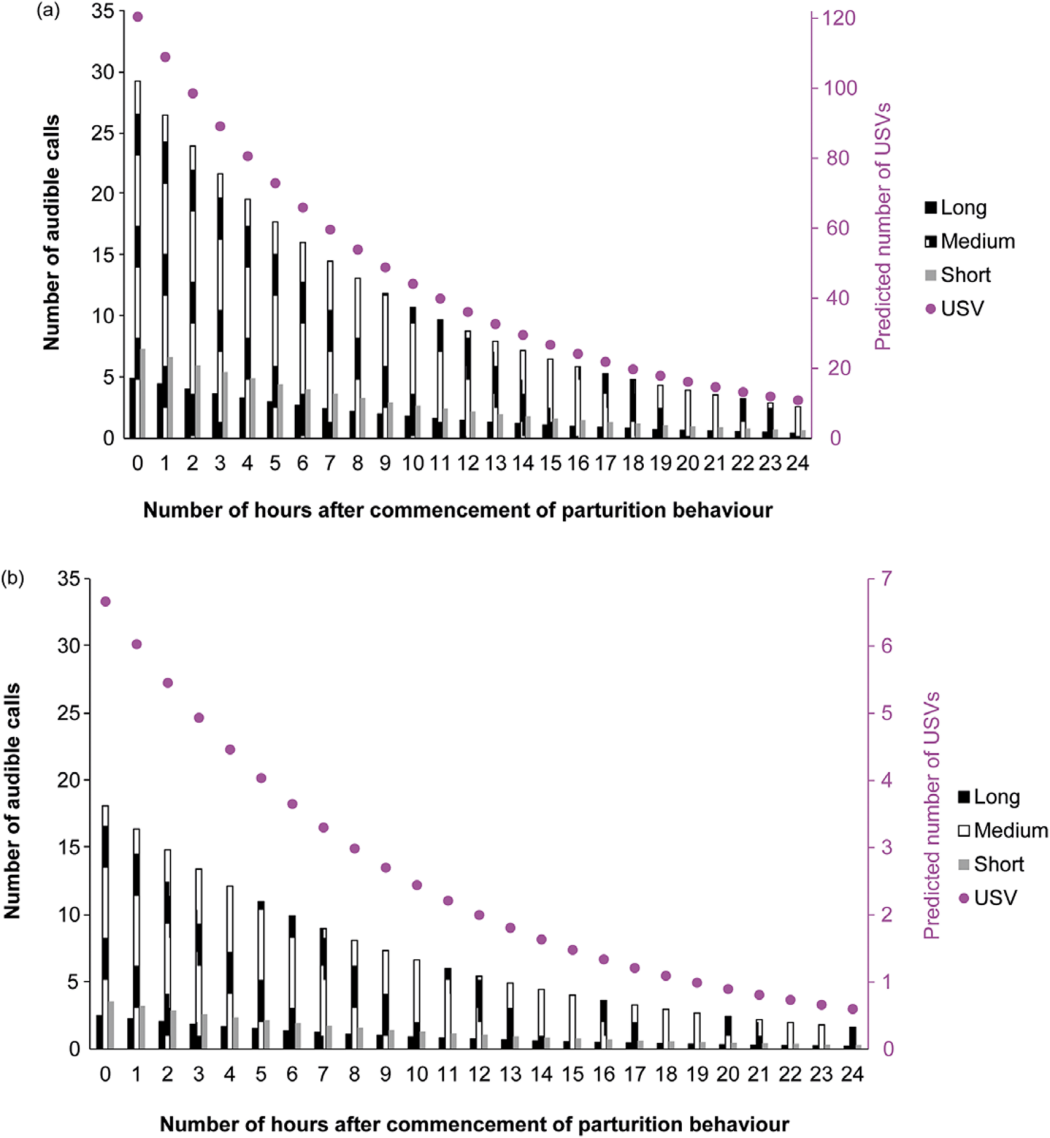
Adjusted number of audible calls and ultrasound vocalisations (USV) per hour interval after the commencement of parturition behaviour (recorded by separate video analysis) in study of C57BL/6J mice (*Mus musculus*). For number of observations per hourly 15-min period and type of call, please refer to Table 1. Figure shows (a) pair-housed and (b) single-housed mice.

Number of audible calls and USVs decreased with the number of hours after commencement of parturition behaviour (X^2^_1, 2,023_ = 6,743.94; *P* < 0.001) in a negative exponential fashion (Figure 5). Both audible calls and USVs were affected by whether mice were single- or pair-housed (X^2^_1, 2,023_ = 5,390.98; *P* < 0.001), type of call (Long X^2^_1, 2023_ = 179.59, Medium X^2^_1, 2,023_ = 502.86, Short X^2^_1, 2,023_ = 95.17; *P* < 0.001), and the interaction between housing configuration (single vs pair housed) and type of call (*P* < 0.001).

Since the number of USVs was also substantial before parturition (see Table 1), their fundamental frequencies were compared between pre-parturient and parturient/post-parturient period and their means are depicted in Table 2 along with their respective standard deviations. Fundamental frequency of USVs was affected by period relative to parturition (X^2^_1,306_ = 8.97; *P* = 0.003) and housing configuration (X^2^_1,306_ = 9.54; *P* = 0.002). Fundamental frequency of USVs was higher in cages with single-housed females (*P* = 0.002) and after the commencement of parturition behaviour (*P* = 0.003), compared to cages with pair-housed mice and the period before parturition, respectively.

**Table 2.** Mean (± SD) fundamental frequency of ultrasound vocalisations (USVs) per housing category

Period relative to parturition	Housing configuration	
	PH	SH
Pre-parturient	62.7 (± 3.7) kHz	63.4 (± 2.4) kHz
Parturient/Post-parturient	63.8 (± 3.7) kHz	65.2 (± 3.5) kHz

SH = Single Housed; PH = Pair-housed mice, in the pre-parturient and parturient/post-parturient periods. No statistical results are presented here.

**Figure 6.**
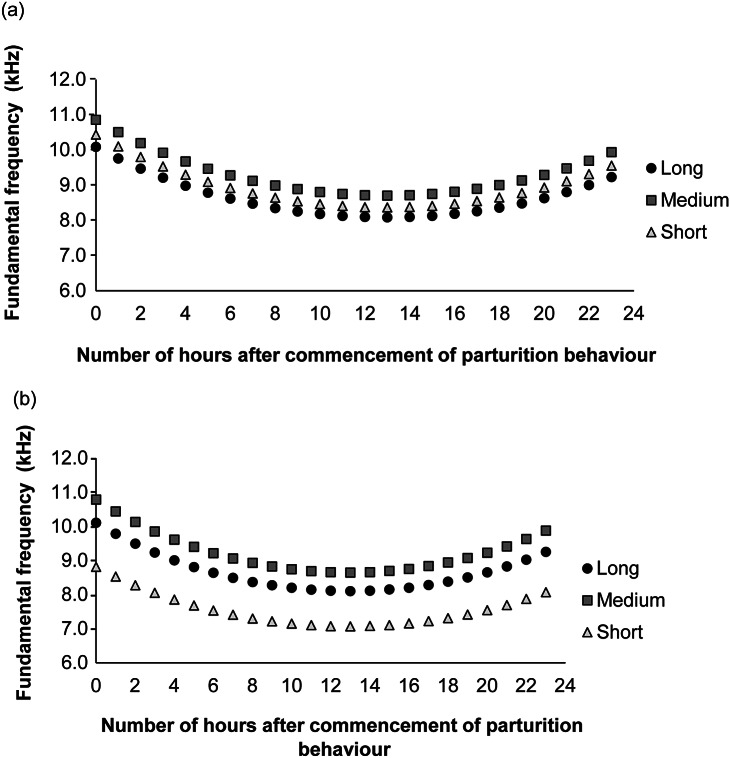
Adjusted fundamental frequency predictions as a function of number of hours post-parturition per type of audible call (Long, Medium, Short) for (a) pair-housed and (b) single-housed C57BL/6J mice (*Mus musculus*).

Fundamental frequency of audible calls was affected by number of hours after commencement of parturition behaviour in a quadratic fashion (X^2^_1,785_ = 16.18; *P* < 0.001), type of call (Long X^2^_1,785_ = 5.73; *P* = 0.017; Medium X^2^_1,785_ = 19.55; *P* < 0.001; Short as reference), housing configuration (X^2^_1,785_ = 10.31; *P* < 0.001) and the interaction the two last variables (PH × Long X^2^_1,785_ = 4.11; *P* = 0.043; PH × Medium X^2^_1,785_ = 6.13; *P* = 0.013; SH and Short as references). The fundamental frequency of Short calls (after commencement of parturition behaviour) was higher in cages with pair-housed compared to single-housed mice (Z = 3.21; *P* = 0.017). For single-housed mice, Medium calls had a higher frequency than Short calls (Z = 4.42; *P* < 0.001). No other differences were found in the fundamental frequency among types of calls. Figure 6 depicts the adjusted fundamental frequencies as a function of the number of hours after commencement of parturition behaviour.

## Discussion

This study with C57BL/6J mice shows for the first time that mouse vocalisations differ strongly between before and after the start of parturition (birth of the first pup) in a way that can be detected under normal housing conditions for breeding laboratory mice. This observation has direct practical relevance, as it suggests that vocalisations could be used to automatically detect parturitions.

The probability of vocalisations is substantially higher after the birth of the first pup compared to prior to parturition, when the likelihood of vocalisations is near a 50% chance of either occurring or not (except for USVs in breeding pairs, as discussed in the next section). Our results are based on an analysis of audio recordings against synchronised video recordings, so that we were able to establish the birth of the first pup independently and more accurately. If these results can be reproduced and validated on a large scale this would mean that time of birth in cages fitted with microphones can be determined more accurately for litters born during times when there are no technicians visually monitoring cages or even present in an animal facility. Knowing that parturition has started makes it easier to watch out for cases of dystocia where intervention may be needed, such as the provision of supportive therapy (Hankenson 2014). Early monitoring of newborn litters of rare or vulnerable genotypes (e.g. Ts1Cje strain, [Ferres *et al.* 2016] or mouse models of Hypomorphic Collagen VII deficiency [Strege *et al.* 2024]) or that exhibit maternal behaviour impairments (Wang & Storm 2011) is also important for improving chances of survival. Reliable detection of parturition improves accuracy of record-keeping in breeding facilities: for example, it decreases the possibility that a parturition with total litter loss goes unnoticed. In previous studies using video recordings we have demonstrated that a substantial proportion of dead pups (more than 50% for trio-housed C57BL/6J breeders) would never be found in standard husbandry conditions as they have already been eaten before the day 1 cage check which is typically the first time a litter is detected visually (Morello *et al.* 2024). Timely detection of parturition is also crucial when exact determination of pup age matters for research applications (i.e. developmental research; Qiu *et al.* 2024). Ultimately, it increases options and accuracy of data collection for further research on maternal behaviour and causes of pre-weaning mortality.

For audible vocalisations, there was no difference between single and pair housing either at pre-partum levels or in the magnitude of the increase after the commencement of parturition. In contrast, there were more USVs in cages with pair-housed breeders both before and after the commencement of parturition. This suggests that USVs are related to communication between the male and female adult in the cage, whereas audible vocalisations are related more directly to parturition (pain) and/or mother-offspring communication. USVs are emitted by males during courtship behaviour (Sales 1972; Whitney *et al.* 1973; Warburton *et al.* 1989; Barthelemy *et al.* 2004), whereas females produce both ultrasonic vocalisations (Neunuebel *et al.* 2015) and broadband sounds (Finton *et al.* 2017) during courtship displays. Pup USVs may also be involved as the female is especially receptive to neonatal USVs during post-partum oestrus (Whitney *et al.* 1973). To our knowledge, there is no published literature on laboratory mouse females vocalising during parturition, but audible vocalisations are known to be produced in situations of pain and distress, such as ear or tail snipping for identification or when being suspended by the tail (Williams *et al.* 2008; Ruat *et al.* 2022). Labour is understood to be painful, due primarily to uterine contractions as well as tissue stretching and distension as the foetuses move through the birth canal (Labor & Maguire 2008). Audible vocalisations may also come from neonatal pups, which emit broadband spectrum signals which are known to inhibit biting and injury by adults (4–40 kHz) (Haack *et al.* 1983) and low-frequency calls (major energy < 10 kHz; frequency range rarely > 20 kHz), often referred to as wriggling calls and which release maternal behaviour, such as licking of the pups (Ehret & Bernecker 1986).

There is a negative exponential relation between the number of calls and the number of hours since the birth of the first pup. This continues throughout the time-period for which we analysed recordings: 0–24 h after the birth of the first pup.

After birth, the dam engages in several pup-directed behaviours such as licking, grooming, nest-building, pup retrieval and nursing and, in indirectly related behaviours, such as eating and drinking (Noirot 1964b). The frequency of the pup-directed behaviours reaches a peak around day 1–2 after birth, and decreases until weaning (Noirot 1964a), as opposed to the frequency of other behaviours such as drinking and eating that increase in frequency as lactation progresses (Priestnall 1972). Whereas pre-natal behaviours are hormonally regulated, the maintenance of maternal behaviour after parturition requires stimuli from the litter, which can include vocal communication, olfactory and thermotactile cues (Noirot 1964a). Although mouse neonates have not yet developed their own hearing, they are able to produce vocal cues to elicit maternal behaviour, as discussed earlier.

Despite the audio data storage systems used underperforming and only transferring 40% of the continuous audio data to a secure cloud-based server, the 15-min scan-sampling per hour method produced a usable dataset for the purposes of this study: to understand whether vocalisations differ between recordings before and after parturition. Nevertheless, the prediction estimates of vocalisations should be interpreted with caution, as those are based on data that represent 25% of each studied hour. Thus, the total number of vocalisations per hour is likely to be higher in reality. Also, the microphone used in this study was selected to enable the recording of both audible and ultrasonic vocalisations and, due to its wide frequency range, had a limited sensitivity for capturing USVs. It is possible, therefore, that USVs emitted further from the microphone’s location within the cage were not all captured. For a more detailed study of type and number of human-audible and ultrasonic mouse vocalisations around parturition, we recommend the use of a continuous sampling method and a microphone with a better sensitivity in frequencies of 40 kHz and above. Future studies should evaluate a longer period before and after parturition, to evaluate whether the increased vocalisation frequency is particular to time around parturition or if it peaks at other times. The present study was carried out with one of the most common strains, C57BL/6J, and before further developing this approach for application as a management tool it needs to be tested with other mouse strains. The cage inspection method where cages are opened if needed to verify the presence of newborn pups is not standard in the facility where the study was carried out, but we have used this method in previous studies in two other breeding facilities without any negative effect on pup survival (Morello *et al.* 2024).

### Animal welfare implications

The findings presented here suggest that automated analysis of vocalisations holds potential to be used as a tool to detect parturition in laboratory mice. Given the limitations of standard management practice to detect new-born pups, the possibility to develop automated tools will have important implications for animal welfare in laboratory mouse breeding. Firstly, this will enable more timely care for the parturient female, providing prompt veterinary support in cases of dystocia. Secondly, it may also improve accuracy in neonatal mortality data. Present practice is insufficient to accurately detect the number of pups born, which leads to mortality rates being underestimated, and the problem of neonatal pup mortality undervalued or even overlooked.
